# Evaluation of superabsorbent polymer (SAP) in oviposition traps used in the integrated control of *Aedes aegypti* (Linnaeus, 1762) and *Aedes albopictus* (Skuse, 1894) (Diptera:Culicidae)

**DOI:** 10.1590/0037-8682-0337-2022

**Published:** 2023-01-23

**Authors:** Marylene de Brito Arduino, Ligia Leandro Nunes Serpa, Osias Rangel, Guilherme Vieira dos Santos

**Affiliations:** 1 Instituto Pasteur do Estado de São Paulo, Laboratório de Biologia e Ecologia de Vetores, Unidade Taubaté, SP, Brasil.; 2 Instituto Pasteur do Estado de São Paulo, Controle de Vetores, Campinas, SP, Brasil.; 3 Universidade de São Paulo, Faculdade de Saúde Pública, Programa de Pós-Graduação em Entomologia e Saúde Pública, São Paulo, SP, Brasil.

**Keywords:** Mosquito trap, Chikungunya, Dengue, Zika, Yellow fever, Hydrogel

## Abstract

**Background:**

Egg collection traps have been studied to assist in the integrated control of arbovirus vectors. Many enhancements have been made over the years.

**Methods:**

This study evaluated the use of a hydrated superabsorbent polymer (SAP) in the egg collection of *Aedes aegypti* and *Aedes albopictus* in ovitraps. An experiment was conducted in the laboratory to determine the minimum concentration of the product to be used in traps in the field to prevent the development of larvae into adults. In the field, the use of polymers has been evaluated using a traditional model of ovitraps. The positive ovitrap index and mean number of eggs per trap indicator were calculated.

**Results:**

In the laboratory, the larvae did not successfully develop to the adult stage, even at the lowest SAP concentration. In the field, the results showed that ovitraps with SAP proved to be effective for egg collection from both species. It was possible to identify sites with the highest concentration of species and expose the ovitraps for a longer period without larval development.

**Conclusions:**

There is a need for studies on the adequacy of this technology for control programs. However, the results showed that ovitraps with hydrogel were potentiated to capture eggs, configuring themselves as another tool for vector control.

## INTRODUCTION

Arboviruses are currently a major and growing public health problem worldwide; dengue, Zika, and chikungunya have afflicted many tropical and subtropical countries with an increasing number of cases. These increases have occurred due to the spread of infestation by *Aedes (Stegomyia) aegypti* and *Aedes (Stegomyia) albopictus* in recent decades^1^ ^-^ ^4^. These species originate from wild areas and adapt to urban environments, where they have developed successfully, especially in artificial containers^5^ ^,^ ^6^.

Research on alternative control methods has helped reduce the use of insecticides and contribute to eco-friendly control practices. Thus, traps for the collection of *Stegomyia* eggs and adults have been studied and applied to detect the presence, evaluate density, and assist in the control of *Ae. aegypti* and *Ae. albopictus* ^7^ ^-^ ^14^. These traps have been used to indicate the onset of infestation and estimate the population density of mosquitoes in the investigated space and time, in addition to being applied to monitor the impact of control measures^15^ ^-^ ^20^.

It is customary to add oviposition attractants to enhance productivity^16^ ^,^ ^21^. Other resources have been added to ovitraps, such as insecticides, microbial larvicides, pheromones, entomopathogenic fungi, and yeast ribonucleic acid (RNA) interference, to hinder the development of immature stages of vector mosquitoes^10^ ^,^ ^22^ ^-^ ^29^.

In light of the growing worldwide interest in superabsorbent polymers, scientists have noted that superabsorbent polymers (SAP) could be a new method of vector management^30^. Thus, studying the addition of SAP to ovitraps could serve as an additional tool in operational mosquito control. Therefore, this study evaluated the use of a hydrated superabsorbent polymer in the egg collection of *Ae. aegypti* and *Ae. albopictus* in ovitraps.

## METHODS

### 1^st^ Phase: Laboratory experiment

Prior to the field experiment, laboratory tests were performed to determine the SAP concentration for use in the field experiment.

The SAP used in this study were applied for agricultural purposes. The product consists of poly (potassium acrylate-co-acrylamide) from polyacrylonitrile (PAN) with high resistance to ultraviolet radiation (UV) degradation and biodegradability, according to the Organization for Economic Cooperation and Development (OECD) Guideline for Testing of Chemicals, item 301B, 1992^31^.

The study was conducted in an insectarium (28 °C ± 2 °C environment; 12 L:12 D photoperiod; 25.5 °C larvae rearing water) and 87% relative humidity (RH) from November to December 2019. The tested doses were determined in a pilot study. Five SAP concentrations were tested (2.5, 5.0, 7.5, 10.0, and 12.5 mg/mL). For this purpose, L_2_ larvae of *Ae. aegypti* were used from eggs collected in the field in the city of São Sebastião, located on the northern coast of São Paulo state, Brazil (23° 45′ 40″ S, 45° 24′ 44″ W). The eggs were immersed in plastic trays containing tap water and decanted for 24hours at a density of 1 egg/10 mL. The water contained TetraMin® feed for tropical fish at a concentration of 0.2 mg/egg (adapted from Gerberg 1994)^32^ as a stimulus for hatching and larval feeding.

Approximately 40 hours after egg immersion, the hatched larvae were pipetted and transferred in groups of seven individuals to 50 mL white plastic cups supplemented with 10 mL of water, with 30 replicates prepared for each SAP concentration and for the control. Labeled transparent plastic cups (250 mL) were used, in which 200 mL of water and TetraMin® were fed per experimental unit, according to the concentration previously described^32^. We added food exclusively on day 0 of the experiment, since the larvae could not be handled because of the use of the gel, after which the SAP dose to be tested was added. Fifteen minutes after the beginning of the experiment, five L_2_ larvae were placed equidistantly in each cup for each of the 30 replicates of the five evaluated concentrations. For the control larvae, only decanted water and the previously mentioned amount of feed were used.

At the same time, each day, the larvae inside the cup were inspected by two laboratory technicians using a stereoscopic microscope, and larval stage and mortality were recorded. The reading followed a system of checking by a laboratory technician rotation, in which the material read by one of the technicians on one day was read the next day by the other technician. This process continued until the end of the experiment, when 100% mortality of the larvae in all cups was achieved.

### 2^nd^ Phase: Field experiment

### Study area

**FIGURE 1:**
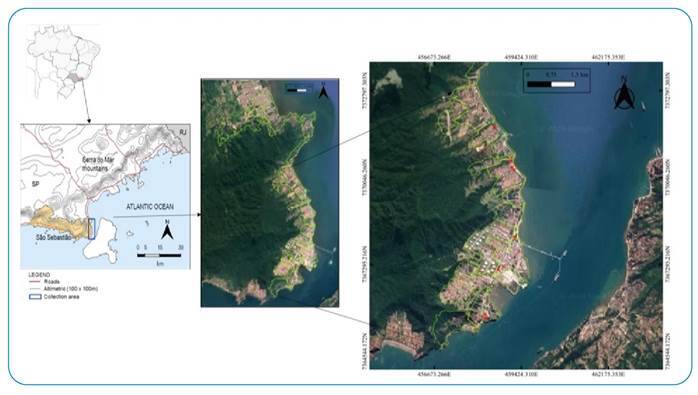
Green: delimitation of the study area. Red: points where traps were installed. São Sebastião, São Paulo state, Brazil.

The field study was conducted in a part of the urban area of São Sebastião from January to August 2020 (Figure 1). The climate is tropical and rainy, with a mean rainfall below 60 mm in the driest months and a mean annual temperature above 30 °C in the warmer months, and not below 18 °C in the coldest months^33^. The region has a high rate of infestation by *Ae. aegypti* and *Ae. albopictus* for more than 20 years^34^, and has experienced annual epidemics of dengue and, more recently, the circulation of Zika and chikungunya viruses.

### Trap description

Traps called ovitraps were used for egg collection in the field, which consist of a black plastic material (10.5 cm in diameter and 15 cm in height) with removable pressed wooden paddles filled with 495 mL of tap water, and 1 mL of beer yeast solution (0.04%) was added to increase attractiveness and stimulate egg deposition by females^20^ ^,^ ^35^ ^-^ ^36^.

### Experimental design

Owing to the time of exposure of the trap in the field, the gel concentration adopted was 15 mg/mL water, slightly above the maximum concentration of the product evaluated in the 1st phase of the laboratory experiment.

**FIGURE 2:**
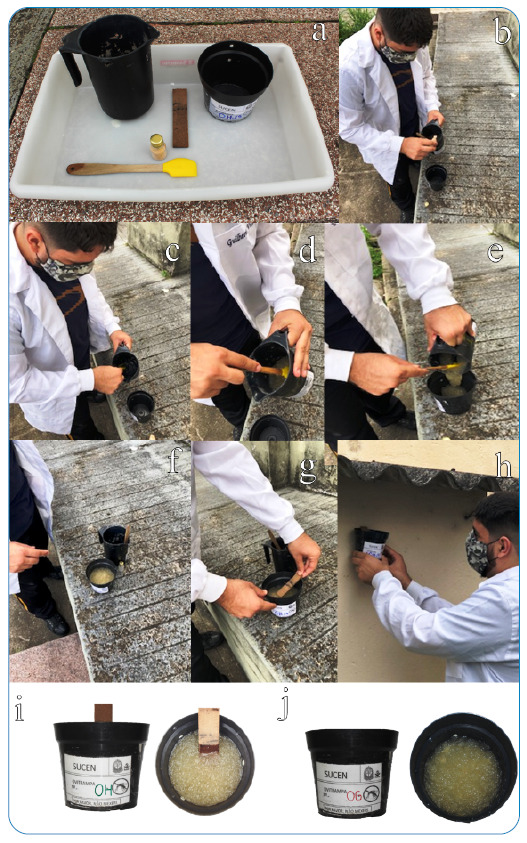
Preparation and installation of traps with gel in the field. The product was mixed in water with a spatula until total hydration and hydrogel formation **(a-g):** installed in a protected site **(h):** frontal view and top view of the OH (Hydrogel Ovitrap) and OG (Gel Ovitrap) **(i-j)**.

*1* ^*st*^ *- Hydrogel ovitrap (OH)*: In addition to water and beer yeast solution (0.04%), SAP (7.5 g) was added to the solution (15 mg/mL). The product was mixed in water with a spatula until total hydration and hydrogel formation (Figure 2a-h), and a pressed wooden paddle was used as a substrate for oviposition. The trap was kept in the field for 5 days as a traditional ovitrap.

*2* ^*nd*^ *- Gel ovitrap (OG)*: The purpose of this trap was to evaluate the performance of the gel under the longest exposure time. The SAP preparation was the same as that described above, without adding attractive or pressed wooden paddles, and the trap was kept in the field for periods ranging from 15 to 38 days.

Fifty traps of each type were installed in a part of the urban area of São Sebastião and in previously selected properties, 50 traps of each type were installed (N = 100). Properties that were frequently positive for *Aedes* spp. during the routine activities of the municipal vector control program were selected, including households and commercial buildings. At each point, properly prepared traps were fixed on or near the walls of the buildings in places protected from rain and at a height of 1.0 to 1.5 meters, with at least 50 m between traps. The traps were installed after the study objectives were clearly explained to the property owners and residents and they agreed to the installation.

### Collection, reading and storage of eggs and larvae

**SUPPLEMENTARY FIGURE 1:**
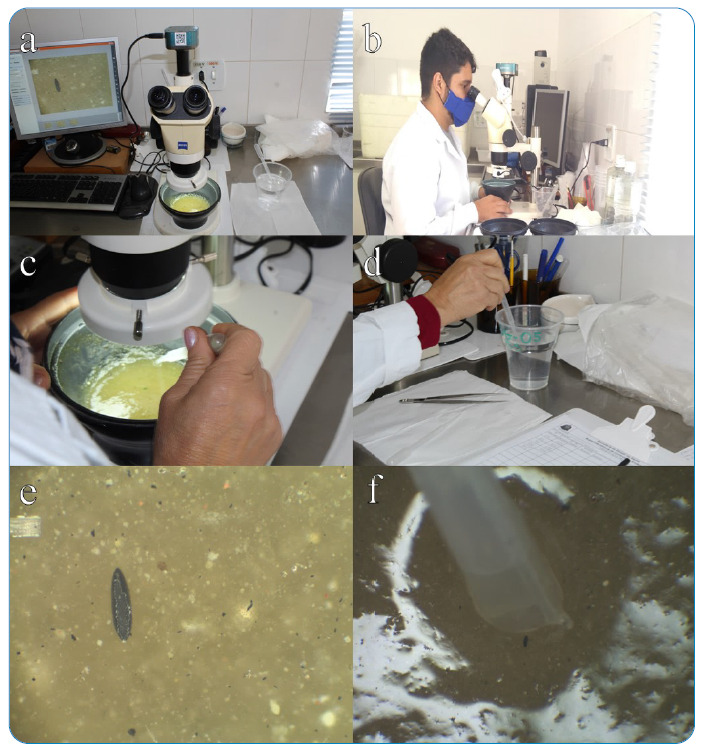
Inspection of the gel in the OHs and OGs under a stereoscopic microscope **(a-b):** collection of eggs and larvae with a pipette **(c-d,f):** Egg laid over the gel **(e)**.

Routine collection of OHs and OGs was previously determined in a pilot study. After the exposure period of each trap type, they were removed, replaced with new ones, and taken to the laboratory, where the gel was inspected while still inside the trap (Figure S1a-b). Eggs and live larvae were removed from the gel by suction with plastic pipettes and transferred to a cup with 200 mL of water with feed, where they remained until they reached the fourth larval stage, at which point they were sacrificed and identified to the species level (Figure S1c-f).

During the pilot study, it was observed that the SAP could be reused because of its ability to dehydrate and rehydrate several times. Therefore, after removing eggs, larvae, and residues from the traps, the gel was rehydrated for subsequent trap reinstallation, a procedure that was repeated for each collection until the end of the study.

OHs were collected in the summer, from February to April, and in winter, from June to August. OGs were exposed from January to August 2020. The hydrogel volume was visually evaluated and categorized according to the following percentages: <10%, 11-20%, 21-50%, and >50% after the exposure time.

All eggs were immersed in water and maintained with TetraMin® tropical fish feed^32^. Larval development was monitored until the fourth stage when the animals were sacrificed and identified.

### Weather and location data

Daily weather data were obtained from the Meteorological Database of the National Institute of Meteorology of the Ministry of Agriculture, Livestock, and Supply of Brazil, collected at the São Sebastião Station, São Paulo, Brazil^37^. The geographic position of each trap was determined by georeferencing each point using a portable global positioning system (Garmin©).

### Data analysis

Nonparametric tests were used in most analyses. Statistica© 12 and R software were used^38^ ^,^ ^39^. In phase 1 of the experiment, the Kruskal-Wallis test was used to compare the time to death of *Ae. aegypti* larvae at different SAP concentrations, followed by the Dwass-Steel-Critchlow-Fligner (DSCF) test (α = 0.05).

In phase 2 of the experiment, *Aedes* (*Stegomyia*) infestation was measured using two indicators: the ovitrap positivity index (POI), which is the ratio of positive traps to installed traps, and the mean number of eggs per trap (MET), which is the ratio of the number of eggs collected to the number of positive traps. In the analysis of these indicators, the Mann-Whitney test was used for each trap type (α = 0.05).

Modeling was performed step-by-step to obtain the models and covariates that best explained the mean eggs per trap type, as well as egg positivity in the OHs. In the first model, the response variable was the arithmetic mean of eggs per day of trap collection, and the covariates were average temperature, rainfall, minimum temperature, maximum temperature, and humidity. Modeling was performed using the GAMMA distribution. The other model is trap positivity. In this model, the response variable was the number of positive traps per day of collection divided by the total number of traps examined using the BINOMIAL distribution, and the covariates were the average temperature, number of eggs, rainfall, maximum temperature, minimum temperature, and humidity. In the modeling process, the class of generalized additive models for location scale and shape (GAMLSS) was used, which was implemented in R by Rigby & Stasinopoulos^40^. For the selection of the final model, the Akaike information criterion^41^ was used, combined with the likelihood ratio test (LRT) and chi-square test for statistical significance values. The goodness-of-fit of the final model was verified by fitting the worm plot and randomness of the residuals according to the Shapiro-Wilk test (α = 0.05).

To evaluate the spatial distribution of the positivity of the two trap types, kernel density curves were plotted for *Ae. aegypti* and *Ae. albopictus* using Quantum GIS (QGIS) 3.16.5^42^.

## RESULTS

**FIGURE 3:**
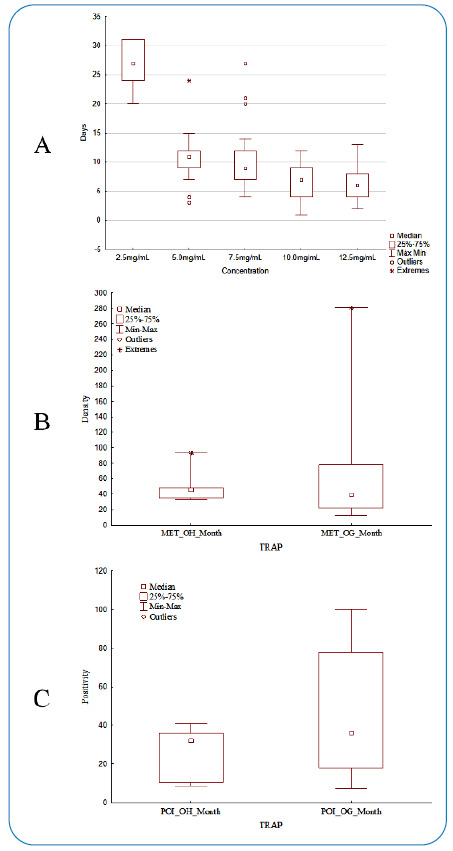
Comparison of exposure time until death of *Ae. aegypti* larvae at the different SAP concentrations tested in the laboratory. Kruskall Wallis test and Dwass-Steel-Critchlow-Fligner test were used **(a):** Monthly mean eggs per trap (MET) distribution according to trap type **(b):** Monthly positive ovitrap index (POI) distribution according to trap type **(c):** for both Mann-Whitney test was used.

In the 1^s^t phase, laboratory experiment, there was a significant difference in larval mortality time (H = 92.52; p = 0.000), except in the comparisons between the 5.0 mg and 7.5 mg (p = 1.000), 7.5 mg/mL and 10.0 mg (p = 0.170), and 10.0 mg and 12.5 mg concentrations (p = 1.000). Overall, the larvae took a longer time to die at the 12.5 mg concentration and a shorter time to die at the 2.5 mg concentration (Figure 3a). The control group had no mortality and reached the adult phase in five days, on average (50% male and 50% female).

**SUPPLEMENTARY FIGURE 2:**
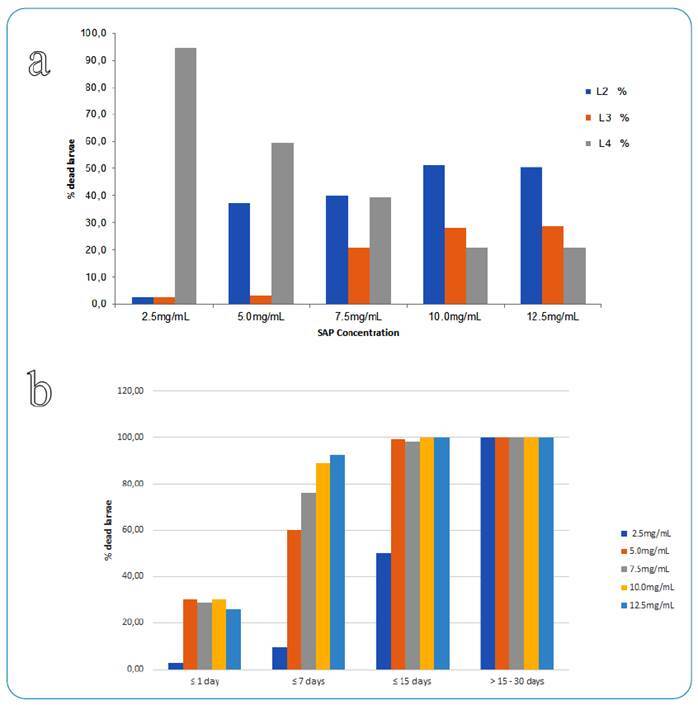
Percent mortality per larval stage for each Superabsorbent Polymer (SAP) concentration **(a):** Overall percent larval mortality according to the SAP concentration and number of elapsed experimental days **(b): L2, L3, L4:** larval stages.

Figure S2a shows that there was an increasing trend in L_2_ and L_3_ mortality as the SAP concentration increased and in L_4_ mortality when the SAP concentration was reduced. Overall, there was an increasing trend in mortality at all tested concentrations up to 15 days of exposure, at which point 100.0% was reached. After day 15, few larvae survived until day 30 (Figure. S2b). The emergence, followed by death, of a single adult female mosquito was observed at the lowest concentration (2.5 mg).

In the 2^nd^ phase, field experiment, the positivity data of the ovitraps are shown in Table 1. During the study period, 1,625 traps were installed and collected:1,250 OHs and 375 OGs. Of the traps installed, 572 (35.2%) were positive for *Stegomyia* eggs. The species *Ae. aegypti* and *Ae. albopictus* specimens were collected from the two trap types. Of the traps containing eggs, 86.4% had *Ae. aegypti*, 5.3% had *Ae. albopictus*, and 6.3% of both species. It is worth mentioning that there was a loss of 25 OG traps throughout the study.

**TABLE 1:** Descriptive analysis of the positivity (POI) and egg density (MET) of *Ae. aegypti* and *Ae. albopictus* by trap type.

		OH Trap	OG Trap	TOTAL
Traps collected	N	1,250	375*	1,625
Total *Stegomyia* eggs	N	14,410	10,805	25,215
Traps positive for *Stegomyia*	N	362	210	572
*Stegomyia* Positivity	POI	29.0	**56.0**	35.2
Confiacce Interval	CI	(26.5 - 31.5)	(50.9 - 60.9)	( 32.9-37.6)
Mean number of *Stegomyia* eggs	MET	39.8	51.5	44.1
Confiacce Interval	CI	(34.7 - 45)	(41.0 - 61.1)	(39.1- 48.9)
Traps with identified species	N	299	149	437
Traps with *Ae. aegypti*	N	281	124	405
	POI	**94.0**	83.2	92.7
Traps with *Ae. albopictus*	N	9	5	14
	POI	3.0	3.4	3.2
Traps with *Ae. aegypti + Ae. albopictus*	N	9	20	29
	POI	3.0	13.4	6.7
Total *Ae. aegypti* eggs	N	8,411	4,182	12,593
Total *Ae. albopictus* eggs	N	194	344	538

**N:** number. *25 traps were lost throughout the study.

Overall, the highest POI (56.0%) and MET (51.5%) were observed in the OGs, although the confidence intervals indicated that MET was similar. The highest POI for *Ae. aegypti* were found in OHs (94.0%).

In the evaluation of the monthly distribution of POIs and METs, no significant difference was observed between the trap types (Figure 3 b e c ).

Rainfall had the greatest influence on the mean number of eggs in the OHs in the initial and final models, followed by the minimum temperature, which was preserved in the final model. Rainfall was negatively correlated with the mean number of eggs, while the minimum temperature was positively correlated (Table S1).

The positivity for OHs was significantly associated with the number of eggs in the initial model; however, it showed a low explanatory contribution (exp(0.0011340) = 1.001135). In the final model, this variable, although interacting with mean temperature, showed little explanatory contribution. Similar behavior was observed for rainfall, which showed an explanatory contribution of approximately 4.7% (Table S2).

**SUPPLEMENTARY FIGURE 3:**
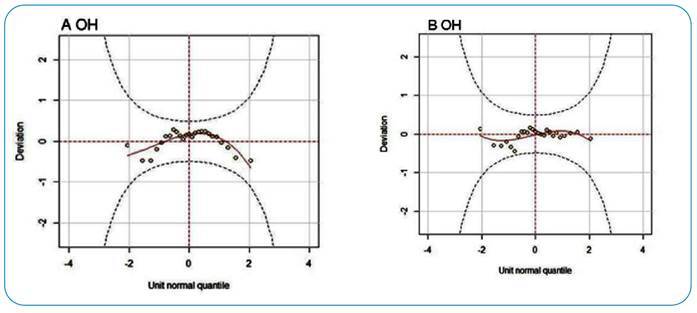
Distribution of the residuals in the worm plot as a function of the analysis of mean eggs **(A):** and positivity **(B):** of the Hydrogel Ovitrap (OH) trap.

In the final model, the number of variables that contributed significantly was higher than that in the model for OHs. The absence of the contribution of humidity in the final model is notable, as is the inclusion of the mean temperature, which in the initial model had a negative value and an odds ratio (OR) = 1.9 in the final model, although the lower confidence interval value was close to zero (Table S2). All the models were adequately fitted (Figure S3).

**FIGURE 4:**
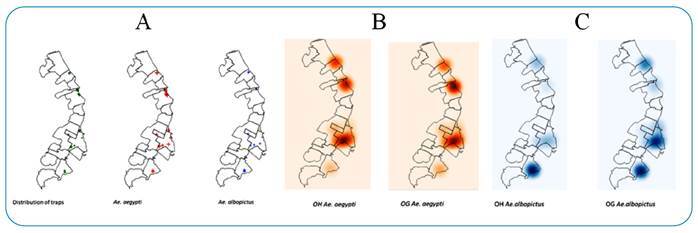
Distribution of trap installation points. *Ae. aegypti* and *Ae. albopictus* clusters **(A):** Spatial distribution of hotspots of *Ae. aegypti* collected in the two trap types **(B):** Spatial distribution of *Ae. albopictus* hotpots in the OH (Hydrogel Ovitrap) and OG (Gel Ovitrap) **(C):** City of São Sebastião, São Paulo, January to August 2020.

Analysis of the spatial distribution of POIs of OHs and OGs revealed that *Ae. aegypti* exhibited the highest number of clusters (Figure 4a). Figure 4b shows the distribution of the intensity of *Ae. aegypti* egg infestations recorded in the two tested trap types. These cluster points are coincident and differ in their degrees of intensity. To the north of the study area, there were intense cluster points of the species in the OGs, whereas the hottest spots of the OHs and OGs were in the south. In both locations, OGs had the most intense hotspots. The spatial distribution of *Ae. albopictus* eggs revealed that the estimated density was significantly higher in the two trap types in the south of the study area, and that the OGs had more hotspots than the other trap types tested (Figure 4c).

The hydrogel remained moist in all the samples at gel concentrations above 10%. At concentrations >50%, larval hatching was observed, as evidenced by broken eggs, although no exuviae were observed.

**SUPPLEMENTARY FIGURE 4:**
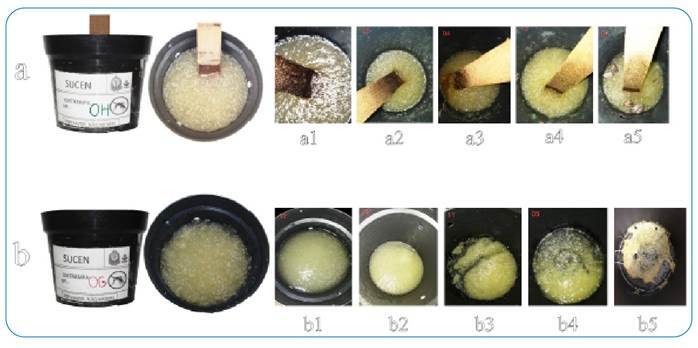
Top view of the ovitrap OH **(a):** and OG **(b):** Aspects of hydrated SAP in the OHs **(a1- a5)** and OGs **(b1- b5)** after exposure in the field.

**SUPPLEMENTARY FIGURE 5:**
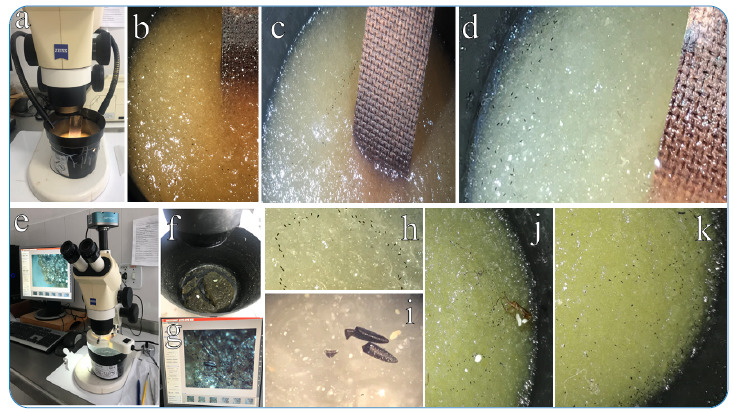
Examination of traps for collecting eggs trapped in hydrogel. Hydrogel Ovitraps-OH **(a-d):** Eggs that were deposited directly on the gel despite the presence of the wooden paddle. Gel Ovitraps-OGs **(e-k):** Whole eggs, despite the gel being quite dehydrated due to exposure time. Eggs broken and trapped in the gel, with no live larvae present.

Representative images of the gel volumes of the OHs and OGs are shown in Figure S4. In the OHs over the five days of trap exposure, partial reductions in the gel volume and stick moisture (a1-a5) were observed. In the OGs with exposure times of 15-38 days, there was a marked reduction in the gel volume, reaching total dehydration in some cases with longer exposure times (b1-b5). Inspection of the traps revealed that, in the OHs (Figure S5a-d) and OGs (Figure S1c-f and Figure S5e-k), eggs were deposited directly on the gel.

## DISCUSSION

Given the constant search for better sustainable measures for the control of arbovirus vectors^7^ ^-^ ^29^, the present study provides important information on the use of SAP in ovitraps. The addition of hydrated SAP to the traditional ovitrap proved operationally efficient. The use of gel was found to be an impediment to the complete and successful development of *Ae. aegypti* and *Ae. albopictus*. The results presented here add another tool for the control of urban arbovirus vectors. They allow the ovitrap to stay longer in the field, increasing capture time without larval development.

The use of hydrogel in the trap confirmed the presence of two species, *Ae. aegypti* and *Ae. albopictus* ^34^. The large number of eggs deposited on the gel of the OH and OG traps demonstrated that the hydrated SAP stimulated the deposition of eggs directly on the hydrogel (Figure S5). Barrera et al. (2013)^12^ showed that the *Ae. aegypti* readily deposited eggs on a polyacrylamide (PAM) hydrated gel with hay infusion and water.

The egg positivity for *Ae. aegypti* and *Ae. albopictus* observed in both types of traps supports the claim that the use of hydrated SAP in traps is efficient for the detection of both species^20^.

We believe that the gel did not exert its greatest attractiveness and stimulation effects on females in OH after five days in the field, which is a limitation of the present study. The OGs showed proportionally higher egg density, although they did not receive the attractant, which is explained by the fact that the water was “encapsulated” in the SAP particles, which possibly compromised the release of the attractant in OH, as more is released when in liquid form due to the fermentation of the attractant in traditional ovitraps. Conversely, the OGs showed a proportionally higher egg density, although they did not receive the attractant, likely because they remained exposed for a greater number of days between collections. This result shows that it is possible to increase trap productivity by adding the gel. The use of this product in egg traps allows the adoption of a period of exposure longer than the conventional period without the risk of egg hatching and cycle development.

Even when using a volume of 500 mL of water was used for hydration of the gel in the traps, the exposure of the OGs for more than four weeks did not affect the humidity of the hydrogel, given that the volume remained above 10.0% in most traps and the females continued to lay their eggs. Barrera et al. (2013)^12^ found no variation in the number of eggs between 40% and 100% gel hydration. Changes in the oviposition behavior of *Ae. aegypti* were reported in other studies, where egg deposition directly on water was suggested to be related to climatic factors, especially when the substrate moisture was below approximately 50.0%^43^ ^,^ ^44^.

Regression models represent the possibility of evaluating isolated indices or models with explanatory variables, especially climatic variables^14^. In the present study, rainfall and minimum temperatures contributed to explaining the mean number of eggs in the final model for OH. Rainfall contributed negatively, whereas minimum temperatures contributed positively. Regarding the model for positivity, the variable number of eggs, without interaction, had the greatest positive contribution to explaining the positivity of the OH traps. This variable contributed positively to the model as an isolated variable and negatively when interacting with minimum temperature. Tropical countries generally experience high temperatures, humidity, and rainfall throughout the year, especially in coastal areas. Thus, it was possible to deduce that in the final model, in the occurrence of interactions with low explanatory contributions, there were changes in parameter values. This demonstrates the need to improve models to establish associations between climatic variables and oviposition indicators, particularly in countries such as Brazil.

Larval hatching was observed in traps with concentrations above 50.0%, as evidenced by the presence of broken eggs on the gel. However, in this situation, no exuviae from dead or newly hatched larvae were found, indicating an unsuitable environment for larval development, which greatly reinforces the possibility of using hydrated SAP as a vector control. Barrera et al. (2013)^12^ also observed that all larvae died when desiccated on the PAM gel shortly after hatching. The authors suggested that this gel could serve as an autocidal oviposition substrate for *Ae. aegypti* if used in ovitraps, independent of the adult trap type, which is what they used.

Because of the positivity and density of eggs in the traps, we can say that the inclusion of hydrated SAP in the traps for oviposition of *Aedes* (Stegomyia) females was efficient in detecting *Ae. aegypti* and *Ae. albopictus*, as well as sites with higher densities. The replacement of water by hydrogel allowed for a longer exposure time of the trap in the field without larval development. This technology could be of great value as an additional complementary tool for surveillance and vector control. Nevertheless, more studies are needed to evaluate the impact of this technology on the vector population and financial costs, in addition to the size and number of traps required per area.

## SUPPLEMENTARY MATERIAL

**SUPPLEMENTARY TABLE 1:** Estimates of parameters regarding the mean number of *Aedes* eggs in hydrogel vitraps (OHs) as a function of climatic variables.

Parameter	Estimate	Standard error	***t* value**	***p* value**
Intercept	2.41965	8.98473	0.269	0.7908
Mean temperature	-1.60666	1.23854	-1.297	0.2109
Rainfall	-0.11696	0.04122	-2.837	0.0109 *
Maximum temperature	0.7322	0.51586	1.538	0.1415
Minimum temperature	0.87862	0.65210	1.347	0.1946
Humidity	-0.03087	0.09262	-1.333	0.7428
Initial model: AIC = 173.51 [Mean eggs ~ Mean T + Rainfall + Max T + Min T + Humidity]				
R^2^ = 0.37				
Residuals: Shapiro-Wilk W = 0.8722; *p* value = 0.0048							
Final model: AIC = 170.23 [Mean eggs ~ Min T + Rainfall]				
R^2^ = 0.29				
Residuals: Shapiro-Wilk W = 0.9374; *p* value = 0.1291				
Variables that contributed most to the final model				
	**DF**	**AIC**	**LRT**	**Pr (Chi)**
		170.23		
Minimum temperature	1	172.21	3.9813	0.046008*
Rainfall	1	175.93	7.6961	0.005534**
Statistical significance	0 ‘***’	0.001 ‘**’	0.01 ‘*’	‘.’ 0.1

**DF:** degrees of freedom; **AIC:** Akaike information criterion; **LRT:** likelihood ratio test; **Pr(chi):** probability(chi-square).

## SUPPLEMENTARY MATERIAL

**SUPPLEMENTARY TABLE 2:** Estimation of the positivity parameters for the number of *Aedes* eggs in Hydrogel Ovitraps (OHs) as a function of climatic variables.

Parameter		Estimate	Standard error	t value	p value
Intercept		-3.1347479	2.9433094	-1.065	0.301
Mean temperature		0.2679809	0.4639732	0.578	0.571
Number of eggs		0.0011340	0.0001516	7.480	6.3e-07 ***
Rainfall		-0.0375834	0.0235392	-1.597	0.128
Maximum temperature		-0.0906159	0.1982585	-0.457	0.653
Minimum temperature		-0.2496704	0.2450850	-1.019	0.322
Humidity		0.0323086	0.0309952	1.042	0.311
Initial model: AIC = 149.3494 - [Rainfall ~ Mean T + Number of eggs + Rainfall + Max T + Min T + Humidity]					
R^2^ = 0.998					
Residuals: Shapiro-Wilk W = 0.97041; *p* value = 0.6555					
Final model: AIC 135.41 [Positivity ~ Mean T + Number of eggs + Rainfall + Mean T: Number of eggs]					
R^2^ = 0.998					
Residuals: Shapiro-Wilk W = 0.98201; *p* value = 0.9219					
Variables that contributed most to the final model					
		**DF**	**AIC**	**LRT**	**Pr (Chi)**
			135.84		
Rainfall		1	140.44	6.5987	0.0102053 *
Mean T: Number of eggs		1	148.07	14.2330	0.000162 ***
		OR	ICI	ICS	
Intercept		0.21626	0.07334	0.63766	
Mean temperature		0.99497	0.93875	1.05455	
Number of eggs		1.00516	1.00490	1.00542	
Rainfall		0.95269	0.91668	0.99012	
Mean T: Number of eggs		0.99982	0.99978	0.99985	
Statistical significance		0 ‘***’	0.001 ‘**’	0.01 ‘*’	‘.’ 0.1

**DF:** degrees of freedom; **AIC:** Akaike information criterion; **LRT:** likelihood ratio test; **Pr(chi):** probability(chi-square); OR odds ratio; **ICI:** lower confidence interval; **ICS:** upper confidence interval.
